# Perioperative application of N-terminal pro-brain natriuretic peptide in patients undergoing cardiac surgery

**DOI:** 10.1186/1749-8090-8-1

**Published:** 2013-01-07

**Authors:** Hua Liu, Chunsheng Wang, Lan Liu, Yamin Zhuang, Xiaomei Yang, Ying Zhang

**Affiliations:** 1 Department of Cardiac Surgery, Zhongshan hospital, Fudan University, No.180 Fenglin Road, Shanghai 200032, China

**Keywords:** N-terminal pro-brain natriuretic peptide, Cardiac surgery, Prognosis

## Abstract

**Background:**

The purpose of the research was to find out the factors which influence plasma N-terminal pro-brain natriuretic peptide (NT-proBNP) levels, then to assess whether preoperative plasma NT-proBNP levels could predict postoperative outcomes of cardiac surgery.

**Methods:**

Between November 2008 and February 2010,225 patients who underwent cardiac surgery in our department were included in the study. The mean age was 61.25 ± 12.54 years, and 156 (69.3%) patients were male. NT-proBNP, CK-MB, cTnT and creatinine levels were measured preoperatively and 24 hours after operation. Postoperatively outcomes including ventilation time, length of stay in ICU and hospital, and mortality were closely monitored. The endpoints includes: 1) use of inotropic agents or intra-aortic balloon pump ≥24 h; 2) creatinine level elevated to hemodialysis; 3) cardiac events; 4) ICU stay ≥5d; 5) ventilation dependence ≥ 72 h; 6) deaths within 30 days of surgery.

**Results:**

NT-proBNP concentrations (median [interquartile range]) increased from 728.4 pg/ml (IQR 213.5 to 2551 pg/ml) preoperatively to 1940.5 pg/ml (IQR 995.9 to 3892 pg/ml) postoperatively (*P* = 0.015). Preoperative atrial fibrillation, NYHA class III/IV, ejection fraction, pulmonary arterial pressure, left ventricle end-diastolic diameter (LVEDD), preoperative plasma creatinine and cTnT levels were significantly associated with preoperative NT-proBNP levels in univariate analysis. The preoperative NT-proBNP was closely related to ventilation time (*P* = 0.009), length of stay in ICU (*P* = 0.004) and length of stay in hospital (*P* = 0.019). Receiver operating characteristic curves demonstrated a cut-off value above 2773.5 pg/ml was the best cutoff (sensitivity of 63.6% and specificity of 80.8%) to predict the mortality within 30d of surgery.

**Conclusions:**

Preoperative plasma NT-proBNP level presents a high individual variability in patients undergoing cardiac surgery. NYHA classification, ejection fraction, pulmonary arterial pressure, LVEDD, atrial fibrillation, preoperative plasma creatinine, and cTnT levels are significantly associated with preoperative NT-proBNP levels. Preoperative NT-proBNP is a valuable marker in predicting postoperative outcomes.

## Background

Brain natriuretic peptide (BNP) is a cardiac hormone released by ventricular myocytes in response to ventricular dysfunction and wall stress. Since it was originally described, BNP has been used mainly in the field of cardiology. The predictive value of NT-proBNP in cardiac surgery has only been assessed in a small number of studies. The main objective of this study was to assess the factors which influence the level of NT-proBNP and the predictive value of NT-proBNP.

## Methods

Between November 2008 and February 2010, 225 patients who underwent cardiac surgery in our department were included in the study. The mean age was 61.25 ± 12.54 years, and 156 (69.3%) patients were male. 85 patients had valve repair or replacement, including mitral valve repair or replacement, aortic valve replacement, tricuspid valve replacement. 105 patients had coronary artery bypass grafting (CABG), including 71 off-pump CABG and 34 conventional CABG. 16 patients had valve replacement or repair concomitant CABG. 7 patients had congenital heart disease repair operation, including atrial septal defect repair, ventricular septal defect repair and Fallot’s syndrome repair. 12 patients had replacement of thoracic aorta. 14 patients had concomitant atrial fibrillation inflation. 154 patients had operations under cardiopulmonary bypass (CPB). The mean CPB and cross-clamp times were 103.31 ± 43.56 min and 54.47 ± 24.37 min, respectively.

Past medical and surgical histories were determined by review of previous records. Preoperative plasma creatinine, CK-MB, cTnT and echocardiogram data were reported. The plasma concentrations of NT-proBNP were obtained before operation and 24 hours after the end of the surgery.

Standardized anesthesia was used in all patients, and each operation was performed through a median sternotomy. Postoperatively all patients had routine care with close monitoring of the cardiovascular system, renal output and other vital signs. Ventilation time, ICU stay time and hospital time were followed.

The clinical endpoints included: 1) use of high doses of inotropic agents or intra-aortic balloon pump ≥24 h; 2) creatinine level elevated to hemodialysis; 3) cardiac events; 4) ICU stay ≥5d; 5) ventilation dependence ≥ 72 h; 6) deaths within 30 days of surgery.

### Statistical methods

Continuous variables are expressed as mean ± standard deviation (SD) or as median and interquartile range(IQR). Categorical variables are shown as numbers(n) and percentages (%). Mann–Whitney U-test was used to compare the results between groups of numerical data and Spearman rank was used for correlations between continuous variables. The utility of BNP as a prognostic indicator of postoperative outcomes was evaluated using receiver operating characteristic (ROC) curves. A p value of <0.05 was considered statistically significant. Data were analyzed using a statistical software program (SPSS for Windows 11.5).

## Results

**Figure 1:**
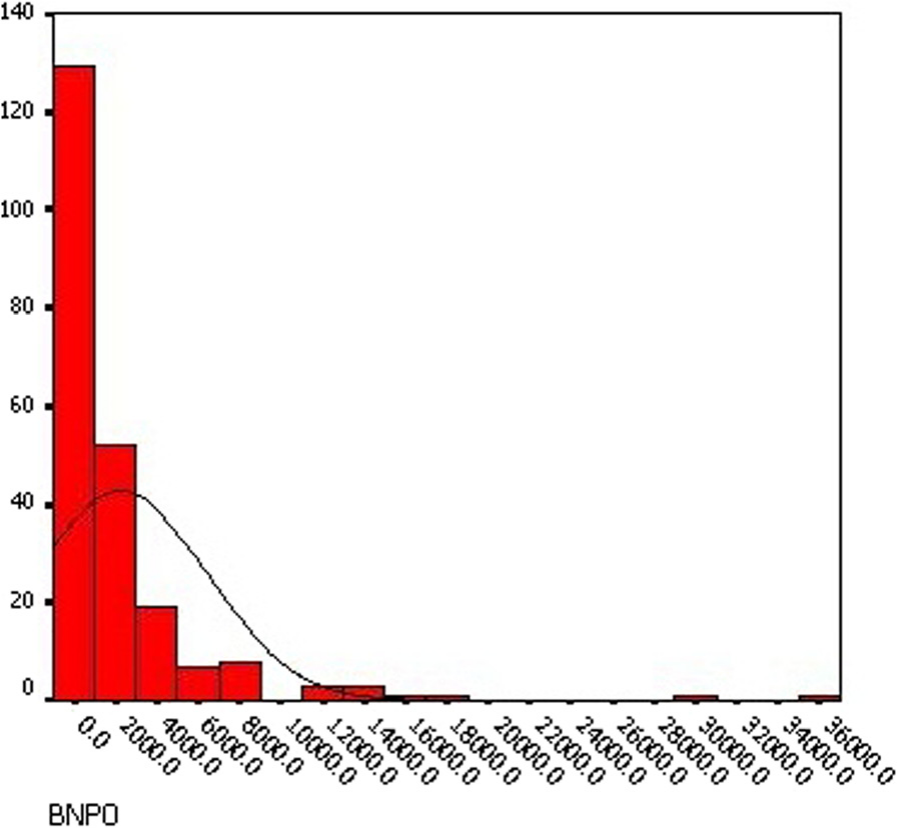
Distribution of preoperative NT-proBNP.

**Figure 2:**
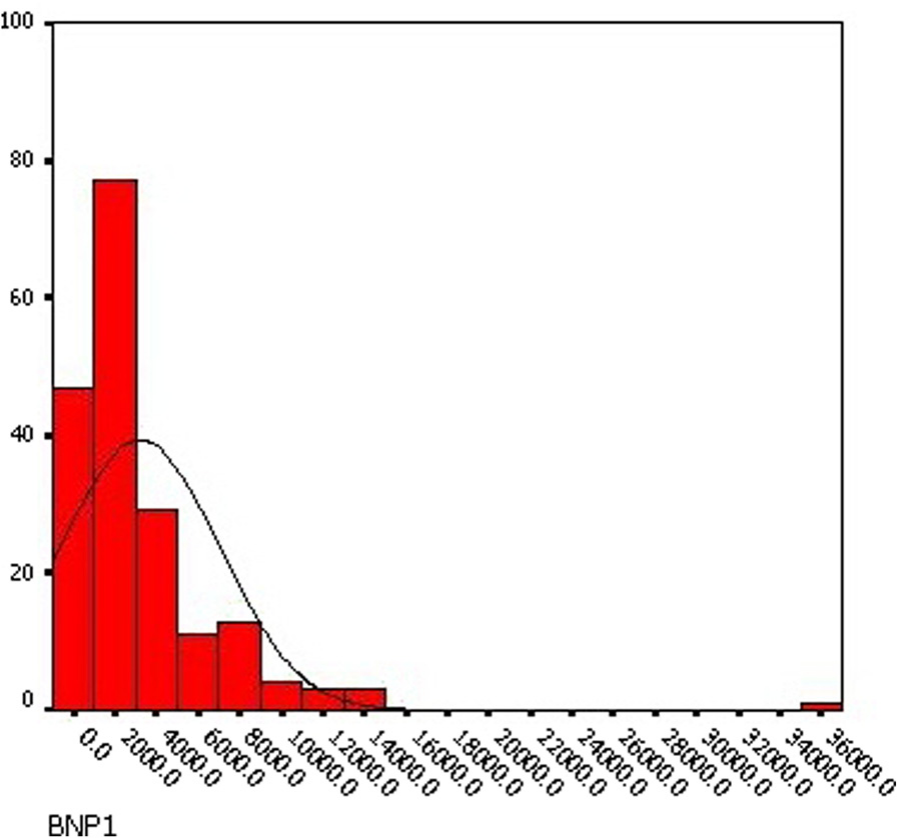
Distribution of postoperative NT-proBNP.

The preoperative NT-proBNP ranged from 19.3 pg/mL to 35000 pg/mL (median 728.4, IQR 213.5 to 2551 pg/mL). The postoperative NT-proBNP ranged from 132.1 pg/mL to 35000 pg/mL (median 1940.5, IQR 995.9 to 3892 pg/mL). Distribution of preoperative and postoperative NT-proBNP level showed in Figure 1 and 2. The difference between the preoperative and postoperative NT-proBNP level was statistically significant (p = 0.015) in paired t-test. Also there was a significant correlation between the preoperative NT-proBNP concentration and the postoperative concentration (r = 0.526, p < 0.001).

The correlation between preoperative patient characteristics and NT-proBNP level were shown in Table 1.Univariate analysis showed that preoperative atrial fibrillation (Af), New York heart association (NYHA) class III/IV, ejection fraction (EF), pulmonary arterial pressure, left ventricle end-diastolic diameter (LVEDD), preoperative creatinine and cTnT were significantly associated with preoperative NT-proBNP levels. Furthermore, patients undergoing valvular surgery had higher NT-proBNP levels compared to patients undergoing CABG.

**Table 1:** The correlation between preoperative patient characteristics and NT-proBNP level

	**Values**	**Median NT-proBNP (g/mL) in two groups**	**r**	***P*-value**
Age (y)	61.25 ± 12.54	-	−0.002	0.982
Female/male	156/69	1048/705.6	-	0.067
NYHA I-II/NYHA III-IV	97/128	277.0/1176.0	-	<0.001
LVEF	0.60 ± 0.11	-	−0.397	<0.001
LVEDD (mm)	56.29 ± 35.93	-	0.290	<0.001
Pulmonary pressure (mmHg)	46.43 ± 17.49	-	0.482	<0.001
Preoperative Cr (μmol/L)	86.66 ± 27.01	-	0.220	0.001
Preoperative CK-MB (IU/L)	20.31 ± 33.23	-	−0.084	0.285
Preoperative cTnT (μg/L)	0.13 ± 0.57	-	0.476	<0.001
Preoperative Af/no	56/169	2148.5/467.6	-	<0.001
CABG/valvular surgery	115/88	436.7/1374.5	-	<0.001

A total of 11 deaths (4.89%) occurred after surgery. Major death cause included postoperative heart failure, neurologic deficit, postoperative renal failure, acute lung failure and sepsis. Median NT-proBNP level in patients with versus without clinical end points was 1998.5 pg/mL and 630.5 pg/mL (p = 0.016), respectively.

**Figure 3:**
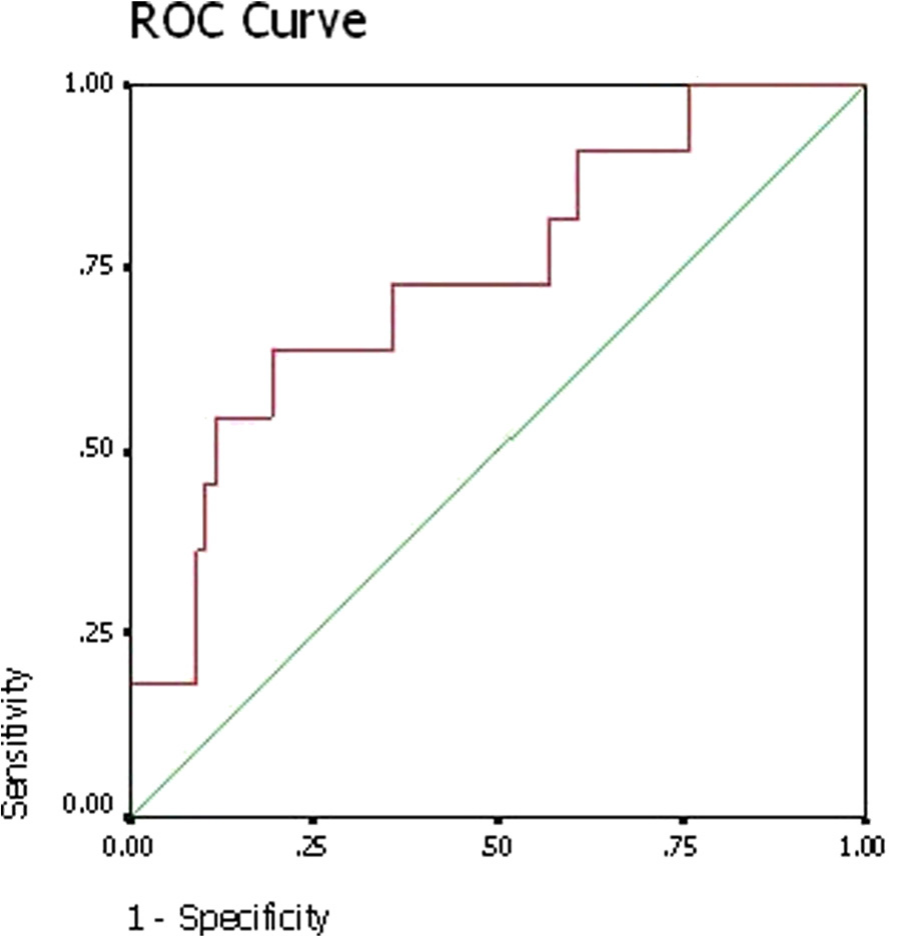
The assessment of the prognostic performance of an elevated NT-ProBNP to predict the mortality within 30d of surgery.

ROC curves demonstrated the assessment of the prognostic performance of an elevated NT-ProBNP to predict the mortality within 30d of surgery (Figure 3). The area under the curve (AUC) was 0.738 (p = 0.008, 95% Confidence Interval 0.58-0.89). A NT-proBNP of 2773.5 pg/ml was the best cutoff (sensitivity of 63.6% and specificity of 80.8%).

In the analysis, the preoperative NT-proBNP showed a significant influence on the need for ICU length of stay (LOS), hospital LOS and ventilation time (Table 2).

**Table 2:** Preoperative NT-proBNP as predictor of surgical outcome

	**Values**	**r**	***P *value**
ICU LOS (d)	3.45 ± 8.17	0.194	0.004
Hospital LOS (d)	19.20 ± 14.44	0.157	0.019
Time of ventilation (h)	21.88 ± 35.19	0.177	0.009

## Discussion

BNP is originally isolated from the porcine brain, although the highest concentration of BNP is actually found in the heart rather than in the brain. It is a cardiac hormone released by ventricular myocytes in response to ventricular dysfunction and wall stress [1]. Probrain natriuretic peptide is a 108 amino-acid propeptide synthesized by myocytes, predominantly from the left ventricle, in response to the increased wall stretch. When released, it is cleaved into 2 fragments: the active brain peptide (BNP) which has the function of natriuresis, diuresis and vasorelaxation [2], and the N-terminal amino acid sequence (NT-proBNP). In rapid time, both BNP and NT-proBNP have emerged as established cardiovascular biomarkers, especially in heart failure, hypertension, acute coronary syndrome or stable ischemic cardiac disease [3]. The main objective of our study was to find out the factors which influence preoperative plasma NT-proBNP levels, then to assess the predictive value of BNP in cardiac surgery.

Steady-state levels of NT-proBNP are as much as four-to six-fold higher than BNP. We used NT-proBNP instead of BNP because of its longer plasma half-life (60-120 min).The longer half-life of NT-proBNP suggests it is more independent of inter- and intra-individual variations [4]. Furthermore, molecules of BNP are unstable at room temperature and start degrading immediately after blood draw if not processed. And in their relation to clinical characteristics and prognostic performance in a large population of patients with heart failure, BNP and NT-proBNP showed subtle differences [5]. This is why we decided to use NT-proBNP in our study.

There is a wide individual variation in plasma NT-proBNP values. As a general guideline, in young, healthy adults, 90% will have BNP <25 pg/ml and NTproBNP ≤70 pg/ml [6]. Our study showed that the preoperative NT-proBNP ranged from 19.3 pg/mL to 35000 pg/mL (median 728.4, IQR 213.5 to 2551 pg/mL). Univariate analysis showed that preoperative atrial fibrillation, NYHA class III/IV, LVEF, pulmonary pressure, LVEDD, preoperative creatinine, preoperative cTnT were significantly associated with preoperative NT-proBNP levels. NT-proBNP had a significant positive correlation with LVEDD, pulmonary pressure, and a significant negative correlation with LVEF and NYHA classification, respectively. This indicates that NT-proBNP is released increasingly from the left ventricle when wall tension or stretch increased as a consequence of volume overload or as left ventricular function deteriorates [7]. Also high NT-proBNP levels are associated with increased creatinine levels and kidney failure. Besides of a reduced renal clearance of NTproBNP, increased cardiac afterload caused by fluid retention is a possible explanation [8]. Experimental and clinical studies have suggested that atrial volume, pressure and wall stretch are the main determinants of the activation of these peptides and are all potentially altered in atrial fibrillation [9], so atrial fibrillation is also a risk factor of increased NT-proBNP. Furthermore, our research found that patients undergoing valvular surgery had higher NT-proBNP levels compared to patients undergoing CABG. We think that patients with valvular diseases had higher heart overload and wall tension than patients with coronary artery diseases.

The postoperative NT-proBNP ranged from 132.1 pg/mL to 35000 pg/mL (median 1940.5, IQR 995.9 to 3892 pg/mL). The difference between the preoperative and postoperative NT-proBNP level was statistically significant (p = 0.015). Previous studies demonstrated that BNP decreases when the aortic clamp is applied and the heart is isolated from circulation. Then there is a minor increase in BNP concentrations following cardiac reperfusion [10]. 24 h post-CPB it arrived its peak value, and returned to the preoperative baseline values 3 weeks after surgery [11]. The postoperative peak concentration of plasma NT-proBNP correlated with the preoperative plasma NT-proBNP concentration (r = 0.526, p < 0.001) in our research. This finding offers a possible explanation why the outcomes after cardiac surgery can be predicted by the preoperative plasma concentration.

A variety of multifactorial risk indexes have been described to help preoperative risk assessment of patients undergoing cardiac surgery. Some studies had focused on the predictive value of preoperative NT-proBNP [12,13]. Hutfless et al. considered that BNP levels > 385 pg/ml could predict the postoperative complications and one-year mortality after heart surgery [14]. Sodeck’s research showed increased levels of NT-proBNP (>647 pg/ml) indicated unfavorable outcome [15]. Grescenzi et al. showed that postoperative NT-proBNP levels are associated with in-hospital mortality and prolonged ICU stay after CABG surgery [16]. Our study demonstrated that a NT-proBNP of 2773.5 pg/ml was the best cutoff (sensitivity of 63.6% and specificity of 80.8%) to predict the mortality within 30d of surgery. In our analysis, median NT-proBNP level in patients with unfavorable clinical end points had significantly higher NT-proBNP level than those without(1998.5 pg/ml Vs. 630.5 pg/ml, p = 0.016). At the same time, the preoperative NT-proBNP showed a significant influence on the ICU LOS, hospital LOS and ventilation time. So NT-proBNP may be become a clinical routine biomarker capable of predicting patients’ perioperative and early postoperative risk because of it is convenient, quick and not expensive. The quantitative cut-off values may be used by physicians in their decision to delay heart surgery in order to further ameliorate the patient. This might be through diuresis, and NT-proBNP level could be quantitatively followed until it reaches more reassuring levels.

## Conclusion

In conclusion, preoperative plasma NT-proBNP level presents a high individual variability in patients undergoing cardiac surgery. The risk factors of preoperative elevated NT-proBNP levels include NYHA classification, EF, pulmonary arterial pressure, LVEDD, Af, preoperative plasma creatinine, and cTnT level. Preoperative NT-proBNP is a valuable marker in predicting postoperative mortality and bad outcome in patients undergoing heart surgery.

## Competing interests

The authors declare that they have no competing interests.

## Authors' contributions

HL carried out the data collection and analysis, drafted the manuscript. CW carried out the artical design and guidance. LL carried out the data collection. YZ carried out the data collection. XY carried out the data analysis. YZ carried out the data collection. All authors read and approved the final manuscript.
